# Assessing the construct validity of cross-national harmonized cognitive assessments using age

**DOI:** 10.1038/s44400-026-00143-2

**Published:** 2026-10-24

**Authors:** Sneha Sarah Mani, M. Maria Glymour, Eleanor Hayes-Larson, Richard L. Jones, Lindsay C. Kobayashi, Zachary J. Kunicki, Kenneth M. Langa, Jinkook Lee, Emma Nichols, Jennifer Weuve, Alden L. Gross

**Affiliations:** 1 Department of Epidemiology, Johns Hopkins Bloomberg School of Public Health, Baltimore, MD USA; 2 Department of Epidemiology and Biostatistics, University of California San Francisco, San Francisco, CA USA; 3 Department of Epidemiology, Boston University School of Public Health, Boston, MA USA; 4 Leonard Davis School of Gerontology, University of Southern California, Los Angeles, CA USA; 5 Department of Psychiatry and Human Behavior, Warren Alpert Medical School of Brown University, Providence, RI USA; 6 Department of Neurology, Warren Alpert Medical School of Brown University, Providence, RI USA; 7 Department of Epidemiology, University of Michigan School of Public Health, Ann Arbor, MI USA; 8 Center for Social Epidemiology and Population Health, University of Michigan School of Public Health, Ann Arbor, MI USA; 9 Institute for Social Research, University of Michigan, Ann Arbor, MI USA; 10 Department of Internal Medicine, University of Michigan, School of Medicine, Ann Arbor, MI USA; 11 Department of Health Management and Policy, University of Michigan School of Public Health, Ann Arbor, MI USA; 12 Institute for Healthcare Policy and Innovation, University of Michigan, Ann Arbor, MI USA; 13 Center for Economic and Social Research, University of Southern California, Los Angeles, California USA; 14 Department of Economics, University of Southern California, Los Angeles, CA USA

**Keywords:** Health care, Medical research, Neuroscience, Psychology, Psychology

## Abstract

Harmonized cognitive measures are key to cross-national comparisons of dementia risk. However, validating such measures cross-nationally is challenging due to differences in cognitive measures and a lack of a reference standard. Age, a characteristic strongly associated with cognition and widely measured, can serve as a benchmark for evaluating how well harmonized cognitive measures are broadly compatible with a common construct. We used cross-nationally harmonized measures from three studies in the US and five studies in England, Chile, Mexico, India, and South Africa (*N* = 27,317). Study-specific estimates of the association between general cognitive scores and age ranged from −0.040 to −0.075 SD units/year of age. The coefficient of variation was 24.1%, indicating moderate relative variability. Results support the hypothesis that cross-nationally harmonized scores, derived from partially overlapping test batteries, measure a broadly similar construct across countries. Future studies should further evaluate criterion, construct, and content validity of cognitive testing in cross-national populations.

## Introduction

The projected rise in the number of dementia cases^1^ poses challenges for quality of life, clinical care, and public health policy, especially in countries where the proportion of older adults is rapidly growing^2^. Of the 152 million older adults projected to be living with dementia by 2050, 75% will be living in low- and middle-income countries (LMICs)^3^, where healthcare and other systems are presently unprepared to meet the complex cognitive needs associated with this demographic transition^4^.

Neuropsychological assessments of cognitive function are essential tools for the characterization of the cognitive status and dementia risk of older adults^5,6^. However, the measurement of cognitive function can be affected by linguistic, cultural, social, and educational contextual factors^7–9^, underscoring a critical need to adopt or adapt, assess, and validate cognitive assessment tools that are appropriate across diverse global contexts^9^. Most existing tools have been developed and validated in high-income countries, posing challenges for cross-national comparability and equitable diagnosis in LMICs^7^.

The Harmonized Cognitive Assessment Protocol (HCAP) is intended to be a cross-nationally comparable, flexible battery of cognitive tests designed to enable researchers to harmonize data on cognitive measures and their risk factors across diverse contexts^8,10^. It is currently implemented in 18 countries^7^, and general and domain-specific cognitive scores have been statistically cocalibrated cross-nationally among countries whose HCAP data are publicly released^8^. These scores are constructed from different test batteries with partially overlapping items, leveraging an item-banking approach to harmonization using item response theory methods. Cross-nationally harmonized cognitive measures are useful in furthering insights into the global etiology of dementia^7^. For example, differences across countries in cognitive functioning can generate new hypotheses about the determinants of dementia. Insights can also be gained by comparing the magnitude of associations between potential risk factors and cognitive outcomes across countries. Evidence triangulation often leverages comparisons of settings with divergent confounding structures to support causal inference^11^. Cross-national comparisons provide promising opportunities for such contrasts.

Cross-cultural validation of harmonized cognitive measures remains a challenge, largely due to the absence of a universally accepted reference standard measurement of cognitive performance^9^. By cross-cultural validation, we refer to similar performance and psychometric properties of harmonized cognitive measures in each country. Measures proximal to brain function, such as markers of white matter hyperintensity or cortical atrophy from MRI and PET imaging, could be putative standards; however, such measurements remain prohibitively infeasible in lower-income countries and may fail to capture the most pertinent cognitive experiences of older adults^5,12^. There are known differences between brain structure and cognitive abilities, hypothesized as cognitive reserve, which complicate direct correlations between brain structure and cognitive functioning^13^.

Alternatively, many cognitive functions are known to decline with advancing aging, and age is universally measured. Age can thus serve as a proxy benchmark for evaluating construct-related validity of harmonized cognitive instruments. Assessing the consistency of the association between age and general cognitive scores may provide evidence as to whether harmonized cognitive scores are broadly compatible with a common underlying construct across diverse populations.

To this end, we estimated associations between age and harmonized factor scores for cognition using data from eight studies that administered either a version of the HCAP, or shorter batteries of cognitive tests. Four studies were from high-income settings, including three US-based studies, and four studies were from LMICs. One of the three US-based studies administered a detailed HCAP assessment that covers multiple cognitive domains, while the remaining two studies administered briefer batteries. The variation among US studies allows us to explore the degree to which battery length affects the age-cognition association, while also providing a within-country benchmark for interpreting the range of age-cognitive score associations observed across countries.

## Results

We first describe the study samples and the observed age–cognition gradients in each population, then estimate study-specific associations between age and harmonized cognitive scores and quantify how much those associations vary across studies. We then report domain-specific associations and supplementary analyses.

### Sample characteristics

**Fig. 1:**
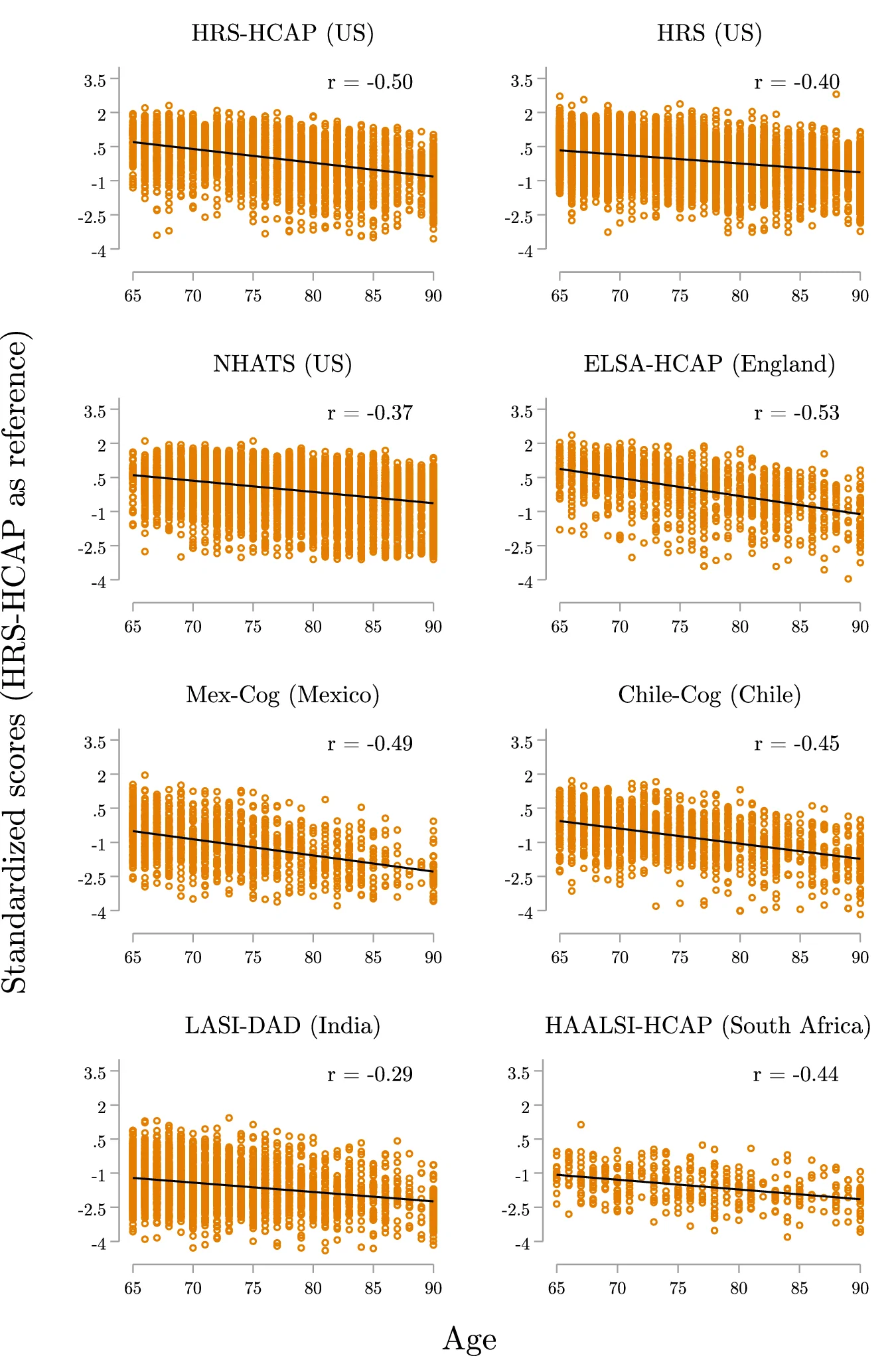
Distribution of general cognitive factor scores by age and study, *N* = 27,317. Weighted Pearson correlation coefficient reported. Data sources: Health and Retirement Study - Harmonized Cognitive Assessment Protocol (HRS-HCAP), HRS 2016 core, National Health and Aging Trends Study (NHATS) - Round 5/2015, the English Longitudinal Study of Ageing-HCAP (ELSA-HCAP), Chile Cognitive Aging Study (Chile-Cog), Mexican Cognitive Aging Study (Mex-Cog), Longitudinal Aging Study in India - Diagnostic Assessment of Dementia (LASI-DAD), and Health and Aging in Africa: A Longitudinal Study in South Africa-HCAP (HAALSI-HCAP).

Table 1 describes the sample characteristics of each study. The mean age ranged from 72.4 years (Longitudinal Aging Study in India Harmonized Diagnostic Assessment of Dementia (LASI-DAD)) to 75.3 years (Health and Aging in Africa: A Longitudinal Study in South Africa -HCAP (HAALSI-HCAP)), and the share of women varied from 49.3% (Longitudinal Aging Study in India Harmonized Diagnostic Assessment of Dementia (LASI-DAD)) to 56.4% (Health and Retirement Study-HCAP (HRS-HCAP)). Scatterplots in Fig. 1 show inverse correlations between age and the statistically harmonized cognitive scores in each study population. Based on visual inspection of the distributions of age and cognitive scores, we assumed a linear relationship between age and cognition in models. The sample-weighted Pearson correlation between age and general cognitive scores ranged from −0.53 in the English Longitudinal Study of Ageing-HCAP (ELSA-HCAP) to −0.29 in LASI-DAD (Fig. 1).

**Table 1:** Summary characteristics of study sample, *N* = 27,318

Study	Age at interview (years)	Female, *N* (%)	Sample, *N*
HRS-HCAP (US)	74.9 (7.2)	2020 (56.4%)	3347
HRS (US)	74.0 (7.2)	5530 (56.3%)	9264
NHATS (US)	74.7 (7.0)	4239 (55.4%)	7333
ELSA-HCAP (England)	74.4 (7.0)	700 (54.0%)	1273
Mex-Cog (Mexico)	73.3 (6.6)	690 (55.5%)	1256
Chile-Cog (Chile)	72.7 (6.3)	848 (55.7%)	1491
LASI-DAD (India)	72.4 (6.6)	1525 (49.3%)	2964
HAALSI-HCAP (South Africa)	75.3 (7.2)	224 (52.7%)	389
Total	74.4 (7.1)	15,776 (56.0%)	27,317

The sample is restricted to ages 65 and above. Estimates for mean age and percentage female include study-specific sample weights.

Data sources: Health and Retirement Study - Harmonized Cognitive Assessment Protocol (HRS-HCAP), HRS 2016 core, National Health and Aging Trends Study (NHATS) - Round 5/2015, the English Longitudinal Study of Ageing-HCAP (ELSA-HCAP), Chile Cognitive Aging Study (Chile-Cog), Mexican Cognitive Aging Study (Mex-Cog), Longitudinal Aging Study in India - Diagnostic Assessment of Dementia (LASI-DAD), and Health and Aging in Africa: A Longitudinal Study in South Africa-HCAP (HAALSI-HCAP).

### Study-specific associations between age and general cognitive scores

**Fig. 2:**
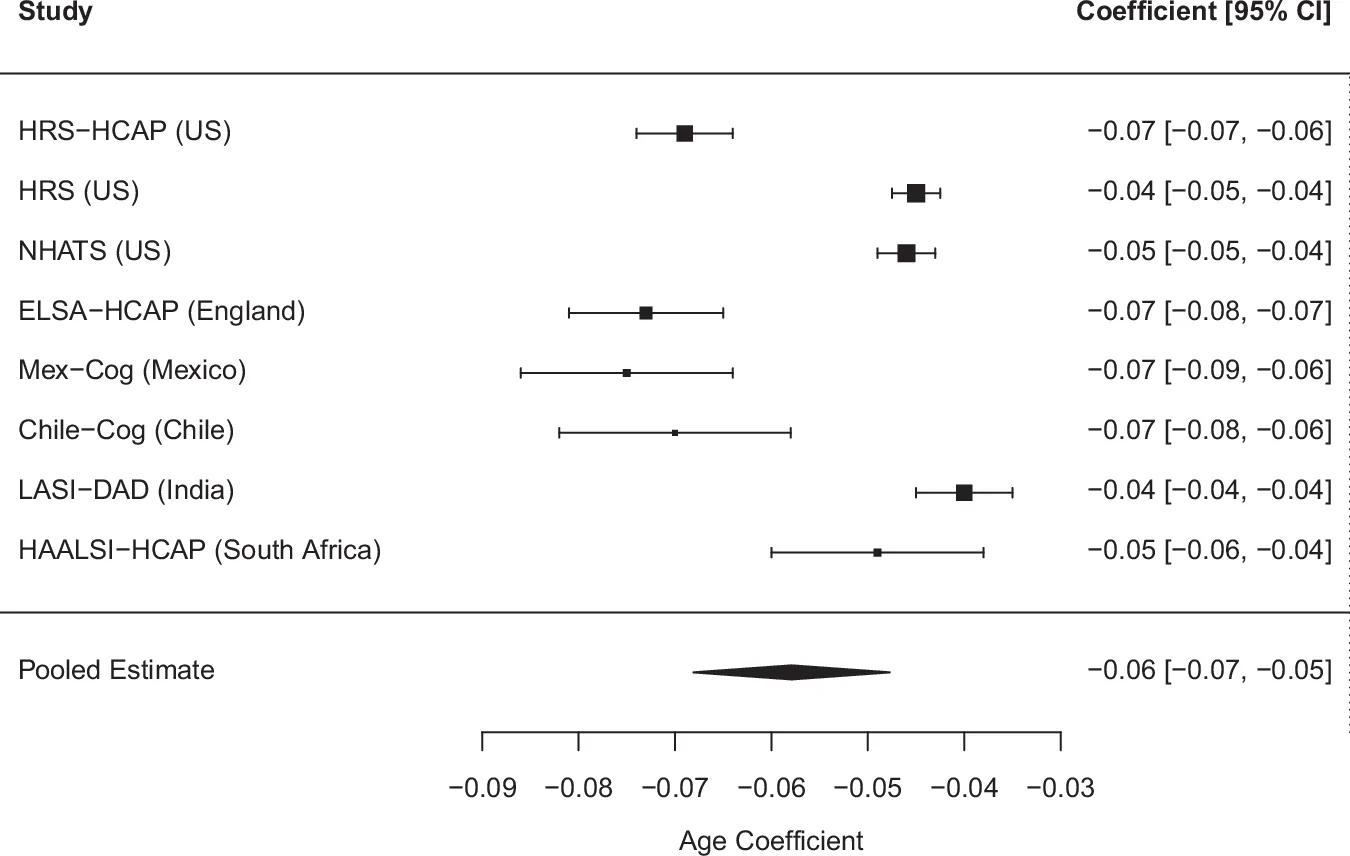
Estimated regression coefficient for age: association between age and general cognition across studies. Associations estimated from linear regression, adjusted for age and weighted by site-specific sampling weights. The associations are coefficients for age, which are directly interpretable as the estimated mean difference in general cognitive score, in SD units, per 1-year increment in age. Data sources: Health and Retirement Study - Harmonized Cognitive Assessment Protocol (HRS-HCAP), HRS 2016 core, National Health and Aging Trends Study (NHATS) - Round 5/2015, the English Longitudinal Study of Ageing-HCAP (ELSA-HCAP), Chile Cognitive Aging Study (Chile-Cog), Mexican Cognitive Aging Study (Mex-Cog), Longitudinal Aging Study in India - Diagnostic Assessment of Dementia (LASI-DAD), and Health and Aging in Africa: A Longitudinal Study in South Africa-HCAP (HAALSI-HCAP).

Model 1 (Fig. 2 and Table 2) presents the regression coefficients for age by study. The estimates ranged from β = –0.040 (95% CI: –0.045 to –0.035) for LASI-DAD to β = –0.075 for Mexican Health and Aging Study-Cognitive Aging Study (Mex-Cog) (95% CI: –0.086 to –0.064). In the US, values were β = –0.069 (95% CI: –0.074 to –0.064) for HRS-HCAP, β = –0.046 (95% CI: –0.049 to –0.043) for NHATS, and β = –0.045 (95% CI: –0.048 to –0.043) for HRS. The Latin American countries had similar estimates: β = –0.075 (95% CI: –0.086 to –0.064) in Mex-Cog and β = –0.070 (95% CI: –0.082 to –0.058) in the Chilean Cognitive Aging Study (Chile-Cog). The estimates adjusting for gender, provided in Table 2, Model 2, were very similar to those not adjusted for gender (Model 1), with the maximum absolute difference between coefficients being 0.003 for HAALSI-HCAP.

**Table 2:** Regression coefficients (β) for age from linear models of general cognitive function

Study	Model 1 (age)	Model 2 (age + gender)	*N*
HRS-HCAP (US)	–0.069 (–0.074, –0.064)	–0.069 (–0.074, –0.064)	3347
HRS (US)	–0.045 (–0.048, –0.043)	–0.045 (–0.047, –0.042)	9264
NHATS (US)	–0.046 (–0.049, –0.043)	–0.047 (–0.049, –0.044)	7333
ELSA-HCAP (England)	–0.073 (–0.081, –0.065)	–0.073 (–0.081, –0.065)	1273
Mex-Cog (Mexico)	–0.075 (–0.086, –0.064)	–0.075 (–0.086, –0.064)	1256
Chile-Cog (Chile)	–0.070 (–0.082, –0.058)	–0.070 (–0.082, –0.058)	1491
LASI-DAD (India)	–0.040 (–0.045, –0.035)	–0.039 (–0.043, –0.035)	2964
HAALSI-HCAP (South Africa)	–0.049 (–0.060, –0.038)	–0.046 (–0.057, –0.036)	389

All models include survey weights.

Model description: Model 1 regresses general cognitive factor scores on age. Model 2 includes a categorical variable for gender. 95% CI provided in brackets.

Data sources: Data sources: Health and Retirement Study - Harmonized Cognitive Assessment Protocol (HRS-HCAP), HRS 2016 core, National Health and Aging Trends Study (NHATS) - Round 5/2015, the English Longitudinal Study of Ageing-HCAP (ELSA-HCAP), Chile Cognitive Aging Study (Chile-Cog), Mexican Cognitive Aging Study (Mex-Cog), Longitudinal Aging Study in India - Diagnostic Assessment of Dementia (LASI-DAD), and Health and Aging in Africa: A Longitudinal Study in South Africa-HCAP (HAALSI-HCAP).

### Cross-study heterogeneity in age–cognition associations

The inverse-variance-weighted coefficient of variation for the association between age and general cognitive scores was 24.1%, indicating moderate relative variability across studies (Table 3). The estimated tau was 0.0141, indicating that approximately 95% of population-specific age coefficients fall within ±0.028 cognitive score units of the pooled estimate (Table 3). The interval between the most extreme beta coefficients was –0.075 and –0.040 (range 0.035). Because this interval is driven by extreme values, it is not directly comparable to τ, which summarizes typical between-study dispersion. That said, the observed range of 0.035 is only about 2.5*τ, suggesting limited between-study spread and a tight dispersion around the pooled estimate.

**Table 3:** Heterogeneity in age-cognition associations by domain

Domain	IV-Weighted CV (%)	τ
General Cognition	24.1	0.0141
Memory	19.6	0.0085
Language	26.8	0.0082
Orientation	36.9	0.013
Executive Function	37.3	0.0156

*NCV* = coefficient of variation (inverse-variance weighted) τ = between-study standard deviation. Both metrics calculated for β coefficients of age from linear regression models across studies. General cognition includes all 8 studies (see Table 2, Model 1). Domain-level results are restricted to HCAP studies (Supplementary Tables 2-5, Model 1).

### Domain-specific associations

Supplementary Fig. 2–5 and Supplementary Tables 2–5 report correlation and regression results for domain-level scores (memory, orientation, and language) from the HCAP studies, showing cross-country patterns broadly consistent with the main results for general cognitive scores. The inverse-variance-weighted coefficient of variation for the association between age and domain-specific ranged was 19.6% for memory, 26.8% for language, 36.9% for orientation, and 37.3% for executive function. The estimated tau ranged from 0.0082 for language to 0.0156 for executive function (Table 3).

### Supplementary analyses

Adjusting the study-specific models for educational attainment attenuated the age–cognition associations in all studies but did not change their direction or the broad cross-study patterns; associations remained negative and statistically significant throughout, with the largest attenuation in Mex-Cog, Chile-Cog, and LASI-DAD (Supplementary Table 6). Additional analysis for the literate and illiterate samples for LASI-DAD, Mex-Cog, and HAALSI-HCAP (Supplementary Table 7) indicates that the regression coefficients for the literate samples were higher than the corresponding coefficients for the illiterate samples in LASI-DAD, Mex-Cog, and HAALSI-HCAP, and were more consistent with coefficients from HRS and ELSA-HCAP (Supplementary Table 7).

Heterogeneity metrics for the education-adjusted associations (Supplementary Table 8) followed a pattern broadly consistent with the unadjusted models: for general cognition, the coefficient of variation was 26.4%, and tau was 0.0124, compared with 24.1% and 0.0141 without adjustment for education. Tau was lower in the education-adjusted models across every domain (memory: 0.0082 vs. 0.0085; language: 0.0074 vs. 0.0082; orientation: 0.0091 vs. 0.0130; executive function: 0.0141 vs. 0.0156), whereas the coefficient of variation increased for memory, language, and executive function and decreased only for orientation (29.7% vs. 36.9%).

In the Bayesian supplementary analysis that incorporated each harmonized score’s standard error of measurement from the item response theory scoring as known measurement error^14^, the age associations were essentially unchanged (Supplementary Table 9), indicating robustness to measurement error in the cognitive scores. (Supplementary Table 9).

## Discussion

We used data from eight studies across six countries to assess the consistency of the association between age and harmonized cognitive factor scores. Findings indicate the downward gradient that cognitive function follows with advancing age, measured from different test batteries with partially overlapping items using item-level calibration, is broadly similar across studies, consistent with our hypothesis that harmonized cognitive scores capture the same underlying dimension of cognitive function. Consistency in the age–cognition association across studies was greatest for memory, the domain built on the most shared item content, such as word-list and story recall, administered in nearly all studies. Domains of orientation and executive function, though rich in items, drew on item sets that had less overlap across studies than memory. To the extent that fewer common linking items threaten harmonization assumptions, correlations of harmonized cognitive scores with age may be more heterogeneous due to extraneous sources of variability. Expansion of shared item content in these domains may be a route to further strengthen cross-national comparability

While all studies showed a consistent negative association between age and general cognition, estimates of the coefficient of variation indicate moderate heterogeneity in the association, which varied by roughly one-quarter of the weighted mean association, consistent with the tight range of estimates relative to tau. No universally accepted threshold exists for interpreting a coefficient of variation of this magnitude, which is a limitation, so values should be interpreted relative to comparable studies and substantive context^15^. We therefore benchmarked the observed 24% coefficient of variation against within-U.S. variability, which was of similar magnitude. This within-country heterogeneity likely reflects differences in cognitive measurement (e.g., battery length, domain coverage) rather than population differences such as sample characteristics, educational factors, or broader contextual factors, providing an internal benchmark for the variability introduced by measurement alone. This benchmark suggests that cross-national magnitude of variation, whatever its specific sources, is comparable to within-country differences. Future work is needed to more firmly establish measurement equivalence across studies. The psychometric framework for evaluating such equivalence is measurement invariance, which tests whether factor structure, item loadings, and item intercepts (configural, metric, and scalar invariance) hold across groups. The harmonization underlying these scores already incorporates part of this logic, linking studies through items assumed to function comparably and examining battery comparability during co-calibration.⁸ Full invariance testing across all eight studies was beyond the present scope but remains an important step, as neither the co-calibration nor the present analysis fully excludes differential item functioning.

Differences in educational attainment also likely contributed to the cross-national heterogeneity we observe. Education is among the strongest determinants of cognitive test performance and varies markedly across the cohorts studied here, in the average and range of attainment as well as in the nature and quality of schooling across educational systems^16–19^. Because education shapes cognitive performance, differences in age–cognition slopes across countries may partly reflect educational differences rather than measurement properties alone^16^. In line with this hypothesis, adjustment for educational attainment attenuated the age–cognition associations in all studies, with the largest attenuation in the Latin American and Indian cohorts. Nonetheless, inferences remained consistent in every study, indicating age captures variation in cognition beyond that attributable to education, while the greater attenuation in some cohorts suggests that educational differences contribute to the cross-national variation in age–cognition slopes. Recalculating heterogeneity for these education-adjusted associations, tau fell in every domain, consistent with education explaining part of this variation; the coefficient of variation, however, rose in most domains. The divergence likely reflects the smaller mean effect used to scale the coefficient of variance rather than growing cross-national inconsistency.

Measurement error in the harmonized cognitive scores does not appear to have substantially biased the age associations. Results from the Bayesian models that incorporated each score’s estimated measurement precision were similar to those that did not. This is consistent with expectation: because cognition is the outcome rather than a predictor in these models, error in the scores would widen residual variation without attenuating the age coefficient^20^. The more consequential source of measurement error is therefore in self-reported age, the predictor, which we consider below.

The lower values of beta coefficients observed in LASI-DAD and HAALSI-HCAP also highlight another limitation regarding measurement error in age. Age is measured through self-report across all surveys, but the precision of self-reported age likely varies across populations. In contexts where birth registration is less systematic among adults from older generations, or where literacy is limited, such as in LASI-DAD or HAALSI-HCAP, individuals may have less precise knowledge of their exact age. This nondifferential measurement error in age could contribute to regression dilution bias^21^. This may partly explain the weaker associations observed in the LASI-DAD and HAALSI-HCAP sample, where more than 55% of participants were illiterate, because measurement error in self-reported age is plausibly greater than in other samples. In LASI-DAD, respondents uncertain of their exact age were asked a series of follow-up questions to approximate their age. Future surveys should flag these respondents to examine whether refinements to this approximation protocol could improve measurement precision.

In conclusion, age-cognition associations were consistently inverse across studies but varied in magnitude, with variability comparable to that observed across studies within the same country. This consistency provides evidence supporting the construct-related validity of these scores. Because decline in cognition with advancing age is among the most robust and widely replicated findings in the field, one should expect a similar age gradient across studies if the scores captured a shared construct. However, it is important to note that consistency with a single criterion such as age offers one form of evidence rather than demonstration of construct validity itself. Since measurement properties and population processes can influence the observed age-cognition association, similarity in coefficients alone cannot uniquely identify measurement equivalence. Nor does consistency with age test whether individual items function equivalently across contexts, which requires formal invariance testing and has been the focus of prior work^8^. While further efforts are needed to establish more robust measures to serve as a benchmark for evaluating the construct-related validity of cross-nationally harmonized cognitive measures, findings presented here provide evidence that cross-national harmonization efforts are broadly compatible with a common cognitive construct, even though cognitive function and its measurement are influenced by multiple contextual factors. Future research could strengthen this validation evidence by triangulating additional criteria known to be related to cognition, such as depression, frailty, and other characteristics^22^, and by testing item-level equivalence directly where harmonized item data allow. Cross-national differences in these scores are best interpreted within the context of this developing body of validity evidence. These neuropsychological assessments remain core and important tools in dementia identification, particularly as ADRD continues to pose a major public health challenge globally.

## Methods

### Study samples

We included *N* = 27,317 participants from six studies that implemented an HCAP to assess cognitive function in older adults in the US, England, Chile, Mexico, India, and South Africa. Between 2015 and 2020, the ELSA-HCAP^23^, Chile-Cog^24^, Mex-Cog^25^, the LASI-DAD^26^, and HAALSI-HCAP^27^, administered HCAP batteries similar to the HRS-HCAP with a goal to facilitate cross-national comparisons of cognitive outcomes in aging populations^10^. While each HCAP study adapted its cognitive test battery to local linguistic and cultural contexts, common cognitive domains and test content were retained to ensure measurement comparability and cross-study test overlap^8^. We also used data from two US-based sources: the 2016 core wave of the HRS and the 2015 round of the National Health and Aging Trends Study (NHATS). Informed consent was obtained from every human participant in all studies, and each study was approved by the appropriate institutional review board. Further detailed information on sampling strategies, eligibility criteria, and fieldwork timelines has been previously published^23–27^.

To ensure all study samples represented a common age range, we restricted the sample to participants aged 65 years and older at the time the survey was administered and with non-missing general cognitive factor scores (Supplementary Fig. 1). Participants aged 90 years and older were top-coded at 90 years to align with the age top-coding procedures used by NHATS and ELSA-HCAP. Top-coding affected 175 participants in HRS-HCAP (5%), 297 participants in HRS (3%), 26 participants in Mex-Cog (2.1%), 40 participants in Chile-Cog (2.7%), 71 participants in LASI-DAD (2.4%), and 19 participants in HAALSI-HCAP (4.9%). This study adheres to the ethical standards of the Declaration of Helsinki. This research relied on publicly available data and was deemed exempt from IRB review.

### Measures

Each study administered a set of cognitive tests, many of which overlapped across studies. Lists of tests, including cross-study overlap for all studies in the present analysis, are provided in Supplemental Table 1. Statistically cocalibrated general cognitive scores were derived using all available tests from each study’s cognitive battery. An item-banking approach was used for the statistical harmonization of factor scores across studies^8^. For the HCAP studies, the test batteries represented multiple domains, including memory, orientation, attention, executive function, and verbal fluency^8^. Further details, including pre-statistical harmonization procedures and analyses of the comparability of the cognitive batteries across studies, are provided in Gross et al. (2023) for HCAPs and in Zhang et al. (2025) for HRS-HCAP alongside NHATS^8,28^. For the present study, in addition to general cognitive scores, we also use harmonized domain-specific factor scores for memory, orientation, language, and executive function from studies that administered an HCAP survey. Self-reported age in years at the time of interview and gender (male or female) were obtained from each study.

### Statistical analysis

We described the characteristics of the sample by country. We plotted the distribution of cognitive factor scores by age and reported Pearson correlation coefficients between age and harmonized general cognitive scores and age for each study. We then specified linear regression models to estimate the association between age and general cognitive scores, stratified by study (Model 1), and additionally adjusted for gender (Model 2). All analyses included study-specific sampling and nonresponse weights, provided by each study, to account for sampling designs and nonresponse. The sampling weights scaled estimates to nationally representative populations of older adults, except for HAALSI, which represents the regional population of the Agincourt subdistrict of Mpumalanga province, South Africa.

We conducted a sensitivity analysis adjusting the study-specific models for educational attainment, as education is among the strongest determinants of cognitive test performance and varies substantially across the included cohorts.²¹ This analysis assessed whether patterns in the association between age and general cognitive scores were robust to differences in educational attainment across populations. Completed educational attainment was categorized according to the International Standard Classification of Education (none or early childhood education; completed primary education; completed lower secondary education; completed upper secondary education; any college)^16^ for all studies except NHATS (no schooling; any level of primary or lower secondary education; completed upper secondary; any college). Zhang et al. (2024) provide more details on the harmonization of education across studies^16^.

We further conducted a sensitivity analysis by stratifying the LASI-DAD, Mex-Cog, and HAALSI-HCAP studies by literacy status, as measurement error in self-reported age may be greater among illiterate respondents. This stratification allowed us to investigate whether the relationships between age and general cognitive scores varied according to literacy status^8^. Literacy status was self-reported in each study by asking respondents whether they could read and write^29–31^.

To assess sensitivity to measurement error in the cognitive scores, we re-estimated each study-specific model incorporating the standard error of measurement of each harmonized general cognitive score, obtained from the item response theory co-calibration, as known measurement error^14^. Using the mi() functionality of the *brms* package^32^, the observed score was treated as a noisy realization of its true value with this imported standard error, and the true value was regressed on age; all models incorporated survey weights. Models were estimated with the No U-turn Sampler (4 chains, 6000 iterations, 2000 warmup), with convergence assessed by R-hat ( < 1.05) and inspection of posterior distributions and trace plots^14^.

We assessed heterogeneity in age-cognition associations across studies using an inverse-variance weighted coefficient of variation ($C{V}_{{weighted}}=\frac{{\sum }_{i=1}^{k}{w}_{i}\cdot C{V}_{i}}{{\sum }_{i=1}^{k}{w}_{i}}$). The coefficient of variation is used to quantify relative variability and measurement reliability. Interpretation of the magnitude of a coefficient of variation is context-dependent; as there are no universal thresholds, prior work has informally categorized values of 20–30% as moderate and >30% as high variability^33^. Because this convention is somewhat arbitrary, we interpret this measure jointly with tau (the between-study standard deviation) and the range of study estimates, rather than in isolation. Larger values of tau reflect greater heterogeneity of associations. Given the small number of studies (*n* = 8), we prioritized tau over I², which is known to be unreliable with few studies^34^. All analyses were conducted using StataSE 17.0 (College Station, TX) and R version 4.4.2.

## Supplementary information

Supplementary Appendix_ R and R.

## Data Availability

All datasets supporting the findings of this study are publicly available. Researchers may access the data through the Gateway to Global Aging Data (g2aging.org) or the official websites of the respective studies, subject to their data use agreements.

## Code availability

Analysis code supporting the findings of this study is available from the corresponding author upon reasonable request.

## References

[1] Livingston, G. et al. Dementia prevention, intervention, and care: 2024 report of the Lancet standing Commission. *Lancet* **404**, 572–628 10.1016/S0140-6736(24)01296-0 (2024). 39096926

[2] Lutz, W., Sanderson, W. & Scherbov, S. The coming acceleration of global population ageing. *Nature* **451**, 716–719 10.1038/nature06516 (2008). 18204438

[3] Nichols, E. et al. Estimation of the global prevalence of dementia in 2019 and forecasted prevalence in 2050: an analysis for the Global Burden of Disease Study 2019. *Lancet Public Health* **7**, e105–e125 (2022). 10.1016/S2468-2667(21)00249-8 PMC8810394 34998485

[4] Albanese, E. et al. Equity in the delivery of community healthcare to older people: findings from 10/66 Dementia Research Group cross-sectional surveys in Latin America, China, India and Nigeria. *BMC Health Serv. Res.* **11**, 153 10.1186/1472-6963-11-153 (2011). PMC3146820 21711546

[5] Glymour, M. M. et al. Will biomarker-based diagnosis of Alzheimer’s disease maximize scientific progress? Evaluating proposed diagnostic criteria. *Eur. J. Epidemiol.* **33**, 607–612 10.1007/s10654-018-0418-4 (2018). PMC7571610 29948371

[6] Craik F. I. M., Salthouse T. A., eds. *The Handbook of Aging and Cognition: Third Edition*. Psychology Press; 2011 10.4324/9780203837665

[7] Kobayashi, L. C. et al. Cross-national comparisons of later-life cognitive function using data from the Harmonized Cognitive Assessment Protocol (HCAP): Considerations and recommended best practices. *Alzheimer’s. Dement.* **20**, 2273–2281 10.1002/alz.13694 (2024). PMC10984496 38284801

[8] Gross, A. L. et al. Harmonisation of later-life cognitive function across national contexts: results from the Harmonized Cognitive Assessment Protocols. *Lancet Healthy Longev.* **4**, e573–e583 10.1016/S2666-7568(23)00170-8 (2023). PMC10637129 37804847

[9] Briceño E. M., et al. Recommended Best Practices for Construct-Centered Cultural Adaptation of the Harmonized Cognitive Assessment Protocol. Published online 2025. 10.1093/geronb/gbaf201 PMC13163222 41138172

[10] Gross A. L., et al. Harmonized Cognitive Assessment Protocol (HCAP): What Makes an HCAP an HCAP? An Adaptive Architecture for Measuring Cognitive Aging Around the World. *The Journals of Gerontology, Series B: Psychological Sciences and Social Sciences*. Published online 2025:gbaf202. 10.1093/geronb/gbaf202 PMC13152205 41128510

[11] Gutierrez, S., Glymour, M. M. & Smith, G. D. Evidence triangulation in health research. *Eur. J. Epidemiol.* **40**, 743–757 10.1007/s10654-024-01194-6 (2025). PMC12304027 40140142

[12] Alber, J. et al. White matter hyperintensities in vascular contributions to cognitive impairment and dementia (VCID): knowledge gaps and opportunities. *Alzheimer’s. Dement.: Transl. Res. Clin. Interventions* **5**, 107–117 (2019). 10.1016/j.trci.2019.02.001 PMC6461571 31011621

[13] Stern, Y. Cognitive reserve in ageing and Alzheimer’s disease. *Lancet Neurol.* **11**, 1006–1012 10.1016/S1474-4422(12)70191-6 (2012). PMC3507991 23079557

[14] Nichols E., et al. Measurement Error and Methodologic Issues in Analyses of the Proportion of Variance Explained in Cognition. *Neuropsychol Rev*. Published online 2024 10.1007/s11065-024-09655-1 PMC12089424 39567460

[15] Cairns, M. & Prendergast, L. A. On ratio measures of heterogeneity for meta-analyses. *Res. Synth. Methods* **13**, 28–47 10.1002/jrsm.1517 (2022). 34328266

[16] Zhang Y. S., et al. Educational Attainment and Later-life Cognitive Function in High- and Middle-income Countries: Evidence from the Harmonized Cognitive Assessment Protocol. *The Journals of Gerontology: Series B*. Published online, 2024:gbae005 10.1093/geronb/gbae005 PMC10997278 38284333

[17] Mani S. S., et al. Own and Parental Schooling, Gender, and Cognitive Performance based on the Harmonized Cognitive Assessment Protocol (HCAP) among Older Chilean Adults. *The Journals of Gerontology, Series B: Psychological Sciences and Social Sciences*. Published online 2025:gbaf140. 10.1093/geronb/gbaf140 PMC12700529 40711851

[18] Angrisani, M., Lee, J. & Meijer, E. The gender gap in education and late-life cognition: Evidence from multiple countries and birth cohorts. *J. Econ. Ageing* **16**, 100232 10.1016/j.jeoa.2019.100232 (2020). PMC8281933 34277334

[19] Nichols E., et al. Exploring the association between higher education and steeper cognitive decline in a nationally representative longitudinal study in India. *Am J Epidemiol*. Published online, 2026:kwag009 10.1093/aje/kwag009 PMC13231860 41536208

[20] Wooldridge J. M. *Econometric Analysis of Cross Section and Panel Data*. MIT press; 2010. Accessed July 27, 2026 https://books.google.com/books?hl=en&lr=&id=hSs3AgAAQBAJ&oi=fnd&pg=PP1&dq=Introductory+Econometrics:+A+Modern+Approach+(or+the+more+advanced+Econometric+Analysis+of+Cross+Section+and+Panel+Data,+MIT+Press,%C2%A02010)+&ots=VZTUmAWZQo&sig=8AtBcv-OiBENfEgPsYS00FWcUZw

[21] MacMahon, S. et al. Blood pressure, stroke, and coronary heart disease: Part 1, prolonged differences in blood pressure: prospective observational studies corrected for the regression dilution bias. *Lancet* **335**, 765–774 10.1016/0140-6736(90)90878-9 (1990). 1969518

[22] Cronbach L. J. Test validation. *Educational measurement*. Published online 1971. Accessed, 2026 https://cir.nii.ac.jp/crid/1572543025365700480

[23] Cadar, D. et al. Cohort Profile Update: The Harmonised Cognitive Assessment Protocol Sub-study of the English Longitudinal Study of Ageing (ELSA-HCAP). *Int J. Epidemiol.* **50**, 725–726i 10.1093/ije/dyaa227 (2021). PMC8271185 33370436

[24] University of Pennsylvania. Harmonized Cognitive Assessment Protocol (Chile-Cog). Published online 2023 https://web.sas.upenn.edu/harmonized-cognitive-assessment-protocol-chile/

[25] Mejia-Arango, S. et al. The Mexican cognitive aging ancillary study (Mex-Cog): study design and methods. *Arch. Gerontol. Geriatrics* **91**, 104210 (2020). 10.1016/j.archger.2020.104210 PMC7854788 32781379

[26] Lee, J., Banerjee, J., Khobragade, P. Y., Angrisani, M. & Dey, A. B. LASI-DAD study: A protocol for a prospective cohort study of late-life cognition and dementia in India. *BMJ Open* **9**, e030300 10.1136/bmjopen-2019-030300 (2019). PMC6677961 31371300

[27] Bassil, D. T. et al. Cohort Profile Update: Cognition and dementia in the Health and Aging in Africa Longitudinal Study of an INDEPTH community in South Africa (HAALSI dementia). *Int J. Epidemiol.* **51**, e217–e226 10.1093/ije/dyab250 (2022). PMC9365629 34871405

[28] Zhang, Y. S. et al. Co-calibration of cognitive performance in the National Health and Aging Trends Study with the Health and Retirement Study’s Harmonized Cognitive Assessment Protocol: Implications for dementia classification. *SSM Popul Health* **30**, 101796 10.1016/j.ssmph.2025.101796 (2025). PMC12138453 40474946

[29] Arce Rentería M., et al. Illiteracy, dementia risk, and cognitive trajectories among older adults with low education. *Neurology*. 2019;93 10.1212/WNL.0000000000008587 PMC6937498 31722961

[30] Strangmann, I. M. et al. The association between multilingualism and cognitive function among literate and illiterate older adults with low education in India. *Alzheimer’s. Dement.: Behav. Socioeconomics Aging* **1**, e70018 10.1002/bsa3.70018 (2025). PMC12814855 41562110

[31] Rosenberg, M. et al. The relationships between cognitive function, literacy and HIV status knowledge among older adults in rural South Africa. *J. Int. AIDS Soc.* **23**, e25457 10.1002/jia2.25457 (2020). PMC7086300 32202047

[32] Bürkner, P. C. brms: An R Package for Bayesian Multilevel Models Using Stan. *J. Stat. Softw.* **80**, 1–28 10.18637/jss.v080.i01 (2017).

[33] Shechtman O. The Coefficient of Variation as an Index of Measurement Reliability. In:SAR, Williams G. M., eds. *Methods of Clinical Epidemiology*. Springer; 2013:39-49 10.1007/978-3-642-37131-8_4

[34] Von Hippel, P. T. The heterogeneity statistic I2 can be biased in small meta-analyses. *BMC Med. Res. Methodol.* **15**, 35 10.1186/s12874-015-0024-z (2015). PMC4410499 25880989

