# Supplementary material for: Assessing the construct validity of cross-national harmonized cognitive assessments using age
Source: NPJ Dement. 2026 Oct 24;2(1):98. doi: 10.1038/s44400-026-00143-2 (PMC13695574; doi:10.1038/s44400-026-00143-2)
Supplement: Supplementary file 1 [file 44400_2026_143_MOESM1_ESM.pdf]

Supplementary Appendix

Supplementary Figure 1: Study participant attrition flowchart across countries and datasets

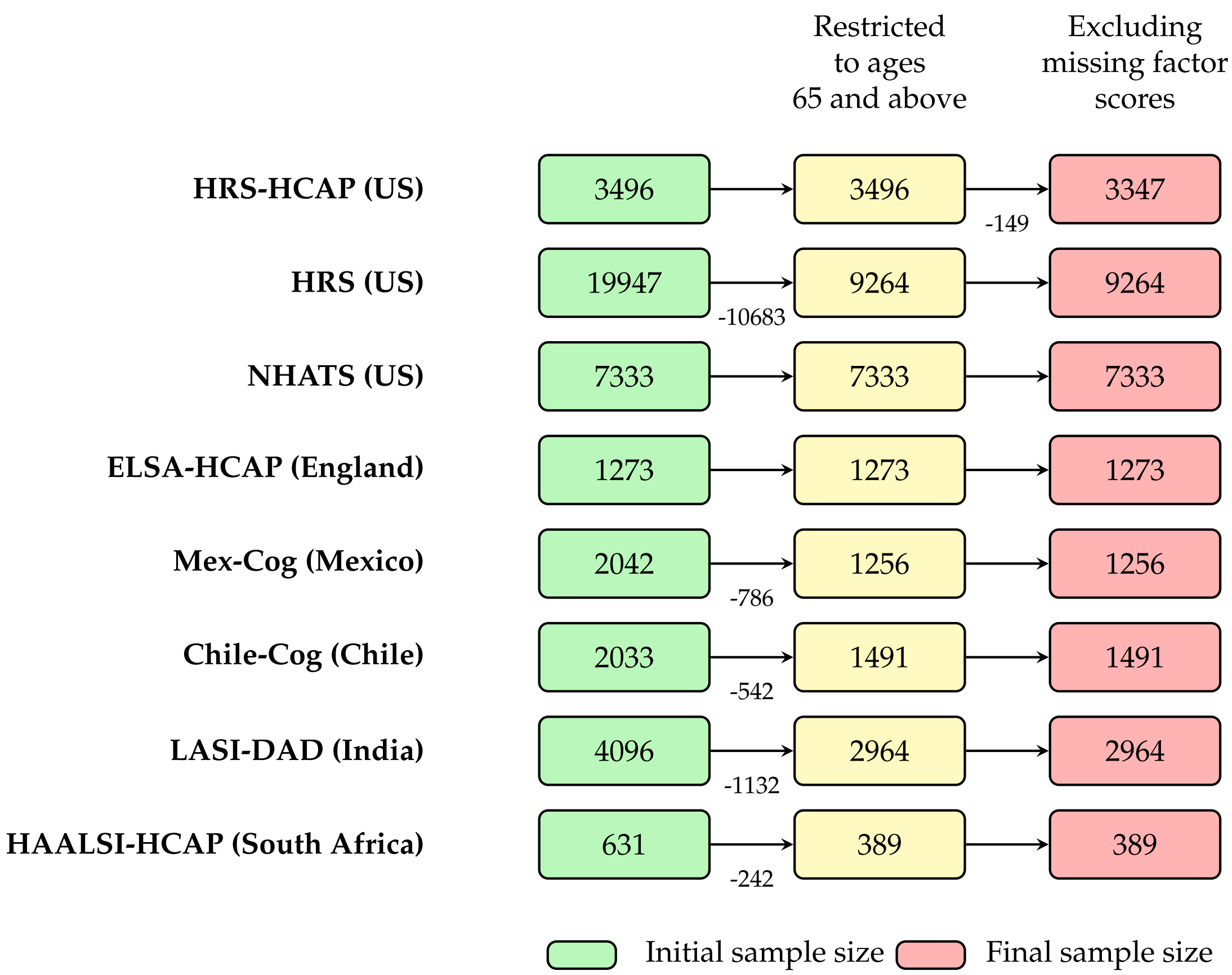

Data sources: Data sources: Health and Retirement Study - Harmonized Cognitive Assessment Protocol (HRS-HCAP), HRS 2016 core, National Health and Aging Trends Study (NHATS) - Round 5/2015, the English Longitudinal Study of Ageing-HCAP (ELSA-HCAP), Chile Cognitive Aging Study (Chile-Cog), Mexican Cognitive Aging Study (Mex-Cog), Longitudinal Aging Study in India - Diagnostic Assessment of Dementia (LASI-DAD), and Health and Aging in Africa: A Longitudinal Study in South Africa-HCAP (HAALSI-HCAP)

Supplementary Table 1: Cognitive test items by domain and study

Item	US (HRS-HCAP)	US (HRS)	US (NHATS)	England	Mexico	Chile	India (literate)	India (illiterate)	South Africa
**Orientation**									
Day of month	√	√	√	√	√	√	√	√	√
Month	√	√	√	√	√	√	√	√	√
Year	√	√	√	√	√	√	√	√	√
Day of the week	√	√	√	√	√	√	√	√	√
What time is it					√				
Where are we					√	√			
What country are we in				√	√	√			√
What state are we in	√				√	√	√	√	√
What county are we in	√			√		√			
What city are we in	√			√			√	√	√$^{\oplus}$
Season of year	√			√		√	√	√	√
Floor of building	√					√	√	√	
Address (street name and/or building number)	√			√		√	√	√	
Name of hospital or district/municipality							√	√	
What is the road or street name?				√					
What is the time?									√
Municipality									√
Where are we sitting right now?									√
**Memory**									
CERAD immediate sum of 3 trials	√	√	√	√					
CERAD immediate sum of 3 trials					√	√	√	√	√
Logical Memory immediate	√			√	√	√	√	√	√
Logical Memory delay	√			√	√	√	√	√	√
Three word delayed recall	√			√	√	√	√	√	√
CERAD word list delay	√	√	√	√					
CERAD word list delay					√	√	√	√	√
CERAD constructional praxis delay	√			√	√	√	√	√	√
CERAD recognition	√			√					
CERAD recognition					√	√	√	√	√
Logical memory recognition	√			√			√	√	√
Brave man delay (East Boston Memory Test)	√			√	√	√	√	√	

Continued on next page

Supplementary Table 1: Cognitive test items by domain and study

Item	US (HRS-HCAP)	US (HRS)	US (NHATS)	England	Mexico	Chile	India (liter-ate)	India (illit-erate)	South Africa
Brave man immediate (East Boston Memory Test)	√			√	√	√	√	√	
Three word immediate registration	√			√	√	√	√	√	√
**Executive functioning**									
Problem solving							√	√	
Ravens progressive matrices	√			√			√	√	√
HRS Number series	√			√					
Trails B time (letters and numbers)	√			√					
Similarities					√	√	√	√	√
Token Test							√	√	√
Numeracy calculations		√							
Number series		√							
Go-No-Go					√	√	√	√	√
Digit Span Forward (single item)							√	√	
Digit Span Backward (single item)							√	√	
CDR calculation-cent									√
Digit Span Forward (multiple items)									√
Digit Span Backward (multiple items)									√
Motor Programming									√
Symbol Digit Modalities Test	√			√					
Backward counting, 100-0	√			√		√			
Backward Day naming							√	√	√⊕
Symbol Cancellation Test					√	√	√	√	√
Serial 3s					√				
MMSE Spelling backwards	√								
Backward counting, 20-0		√			√				
Backward counting, 9-7-5-3-1						√			
Digit Symbol Modalities Test					√	√			
Serial 7s		√		√	√	√	√	√	√
Letter cancellation	√			√					
Trails A time (letters and numbers)	√			√					
Forward day naming									√
**Language**									

Continued on next page

Supplementary Table 1: Cognitive test items by domain and study

Item	US (HRS-HCAP)	US (HRS)	US (NHATS)	England	Mexico	Chile	India (literate)	India (illiterate)	South Africa
Animal fluency	√	√		√	√	√	√	√	√
Name a described cactus	√	√		√					
Name a described coconut							√	√	
What are scissors used for?	√	√		√	√	√	√	√	√
Object naming (watch)	√				√	√	√	√	√
Object naming (pencil)	√				√	√	√	√	√
Name the elbow	√			√	√	√	√	√	√
Write a sentence (or write one's name)	√			√	√	√	√		
Say a sentence								√	
Read and follow command (Close your eyes)	√			√	√	√	√		√
Follow example (close your eyes)								√	
Follow example (raise your hands)						√			
Repetition of a phrase	√			√	√	√	√	√	√
What does one do with a hammer	√			√	√	√	√	√	√$^{\oplus}$
Define Bridge					√	√			
Point to 2 things in the vicinity	√			√	√	√	√	√	√
Where is the local market?	√			√	√	√	√	√	√$^{\oplus}$
Follow 3-stage instruction	√			√	√	√	√	√	√
Name president or Prime Minister	√	√	√	√			√	√	√
Name Vice President		√	√						
Name deputy president									√
Phonemic Fluency									√
Boston Naming Test, uncued									√

Notes: $^{\oplus}$Only administered to respondents who were illiterate. HRS - Health and Retirement Study, HCAP - Harmonized Cognitive Assessment Protocol, NHATS - National Health and Aging Trends Study.
Data sources: HRS-HCAP, HRS 2016 core, NHATS - Round 5, the English Longitudinal Study of Ageing-HCAP, Chile Cognitive Aging Study, Mexican Cognitive Aging Study, Longitudinal Aging Study in India - Diagnostic Assessment of Dementia, and Health and Aging in Africa: A Longitudinal Study in South Africa-HCAP.

Supplementary Figure 2: Correlation between standardized cognitive factor scores for the memory domain and age by HCAP study, N=16,928

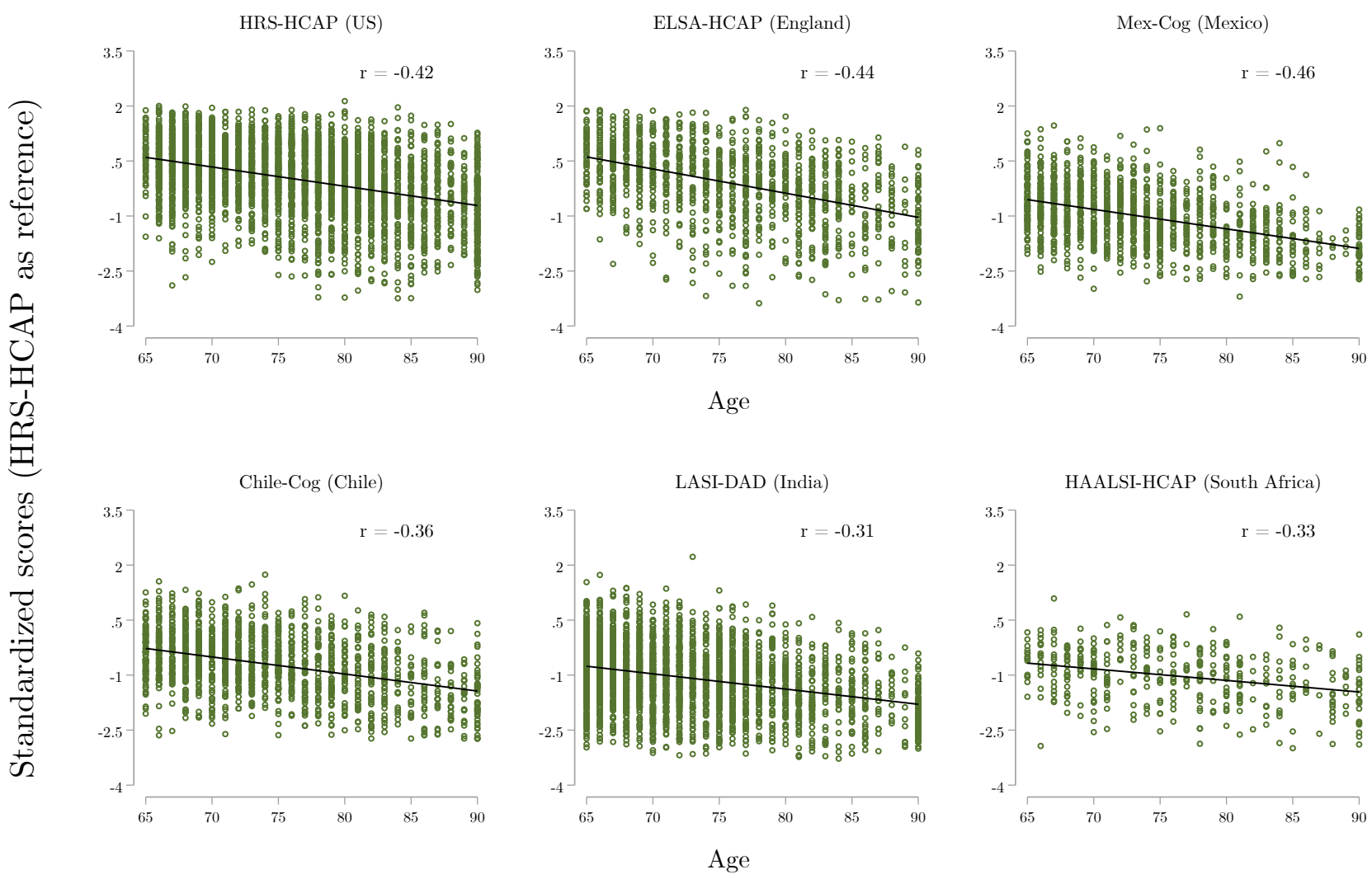

Supplementary Figure 3: Correlation between standardized cognitive factor scores for the language domain and age by HCAP study, N=16,928

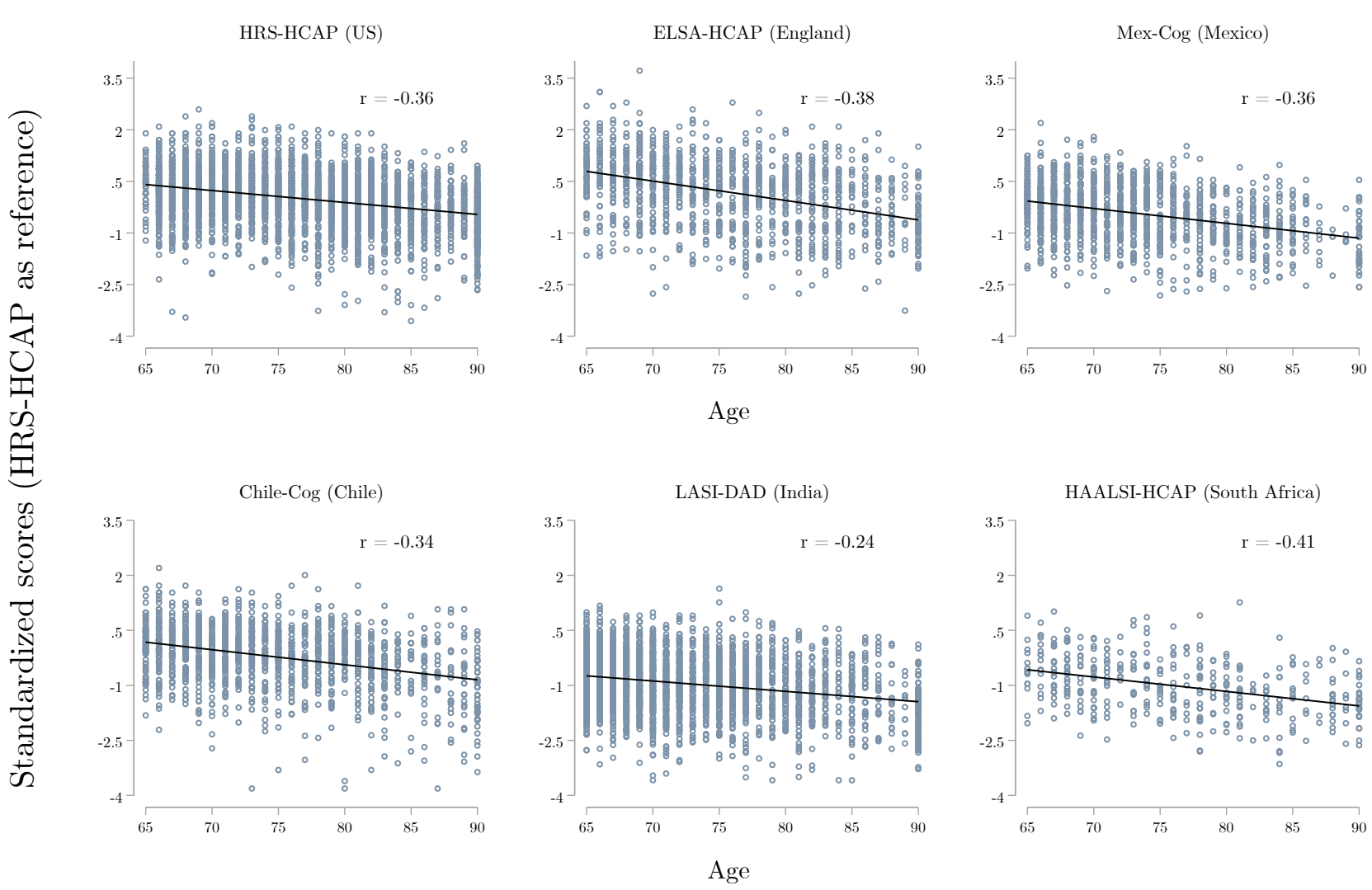

Note: Weighted Pearson correlation coefficient reported.
Data sources: Health and Retirement Study - Harmonized Cognitive Assessment Protocol (HRS-HCAP), the English Longitudinal Study of Ageing-HCAP (ELSA-HCAP), Chile Cognitive Aging Study (Chile-Cog), Mexican Cognitive Aging Study (Mex-Cog), Longitudinal Aging Study in India - Diagnostic Assessment of Dementia (LASI-DAD), and Health and Aging in Africa: A Longitudinal Study in South Africa-HCAP (HAALSI-HCAP)

Supplementary Figure 4: Correlation between standardized cognitive factor scores for the orientation domain and age by HCAP study, N=16,928

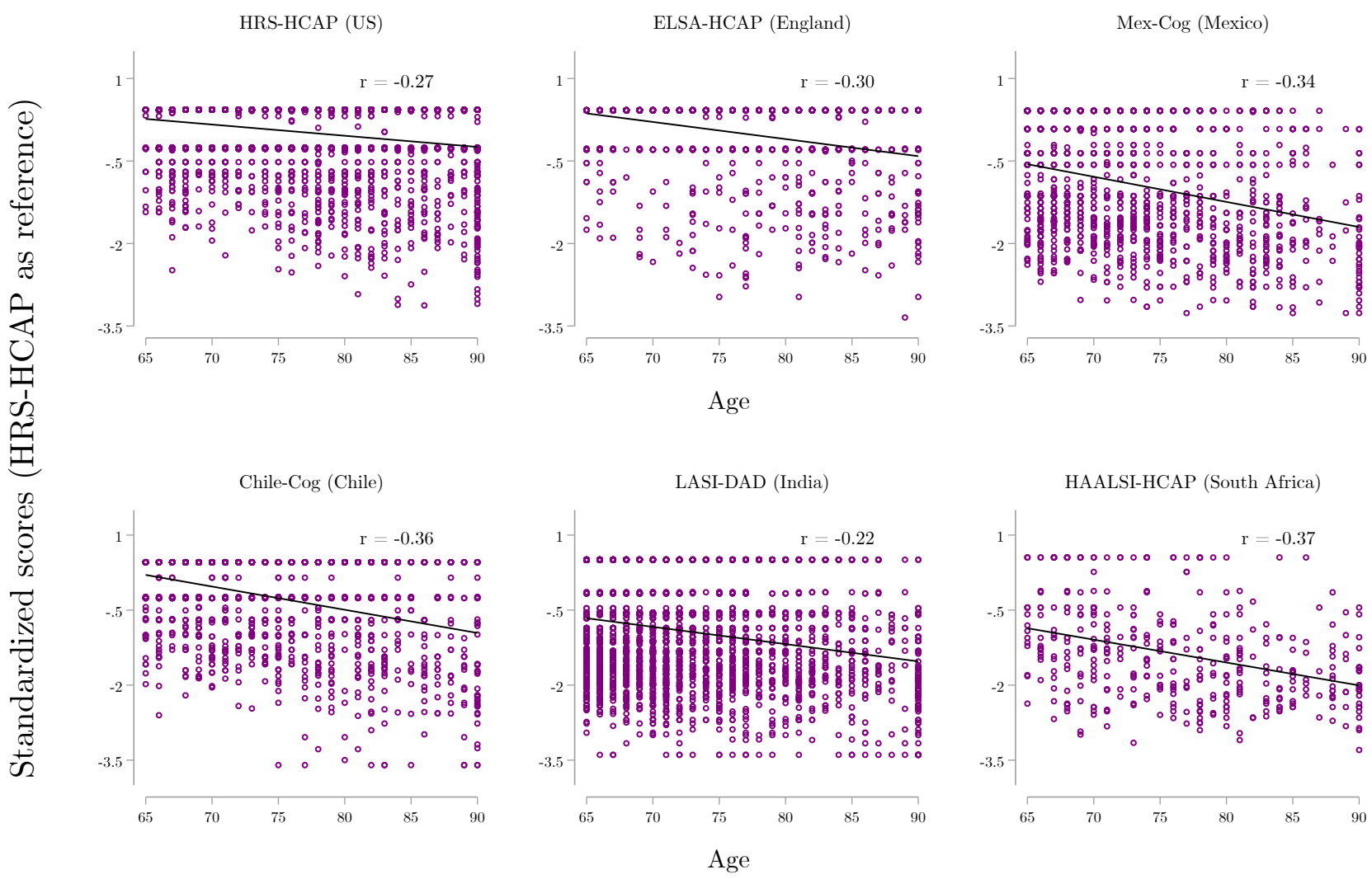

Supplementary Figure 5: Correlation between standardized cognitive factor scores for the executive function domain and age by HCAP study, N=16,928

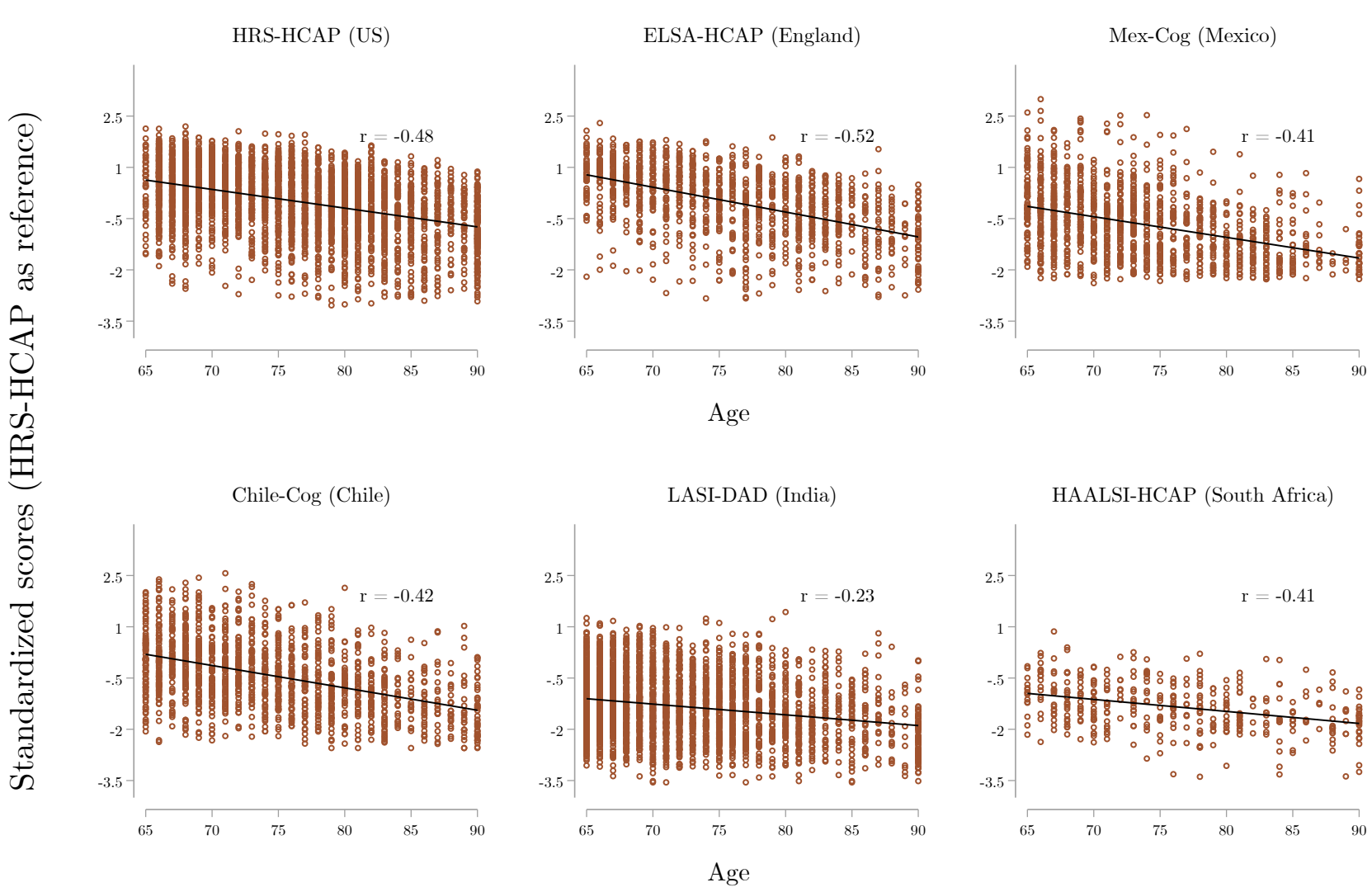

Note: Weighted Pearson correlation coefficient reported.
Data sources: Health and Retirement Study - Harmonized Cognitive Assessment Protocol (HRS-HCAP), HRS 2016 core, National Health and Aging Trends Study (NHATS) - Round 5/2015, the English Longitudinal Study of Ageing-HCAP (ELSA-HCAP), Chile Cognitive Aging Study (Chile-Cog), Mexican Cognitive Aging Study (Mex-Cog), Longitudinal Aging Study in India - Diagnostic Assessment of Dementia (LASI-DAD), and Health and Aging in Africa: A Longitudinal Study in South Africa-HCAP (HAALSI-HCAP)

Supplementary Table 2: Regression coefficients (β) for age from linear models for the memory domain by HCAP study, N=16,928

Study	Model 1(age)	Model 2 (age and gender)	N
HRS-HCAP (US)	-0.055	-0.057	3347
	(-0.060, -0.050)	(-0.062, -0.052)	
ELSA-HCAP (England)	-0.057	-0.058	1273
	(-0.065, -0.049)	(-0.066, -0.050)	
Mex-Cog (Mexico)	-0.054	-0.054	1256
	(-0.063, -0.045)	(-0.063, -0.045)	
Chile-Cog (Chile)	-0.046	-0.047	1491
	(-0.054, -0.038)	(-0.056, -0.038)	
LASI-DAD (India)	-0.040	-0.040	2964
	(-0.044, -0.036)	(-0.044, -0.035)	
HAALSI-HCAP (South Africa)	-0.033	-0.033	389
	(-0.043, -0.023)	(-0.043, -0.023)	

Notes: All models include survey weights.

Model description: Model 1 regresses domain factor scores on age. Model 2 includes a categorical variable for gender. 95% CI provided in brackets.

Data sources: Data sources: Health and Retirement Study - Harmonized Cognitive Assessment Protocol (HRS-HCAP), HRS 2016 core, National Health and Aging Trends Study (NHATS) - Round 5/2015, the English Longitudinal Study of Ageing-HCAP (ELSA-HCAP), Chile Cognitive Aging Study (Chile-Cog), Mexican Cognitive Aging Study (Mex-Cog), Longitudinal Aging Study in India - Diagnostic Assessment of Dementia (LASI-DAD), and Health and Aging in Africa: A Longitudinal Study in South Africa-HCAP (HAALSI-HCAP).

Supplementary Table 3: Regression coefficients (β) for age from linear models for the language domain by HCAP study, N=16,928

Study	Model 1(age)	Model 2 (age and gender)	N
HRS-HCAP (US)	-0.040	-0.040	3347
	(-0.045, -0.036)	(-0.045, -0.036)	
ELSA-HCAP (England)	-0.053	-0.052	1273
	(-0.062, -0.045)	(-0.061, -0.044)	
Mex-Cog (Mexico)	-0.044	-0.045	1256
	(-0.054, -0.035)	(-0.054, -0.035)	
Chile-Cog (Chile)	-0.045	-0.045	1491
	(-0.055, -0.035)	(-0.055, -0.035)	
LASI-DAD (India)	-0.028	-0.027	2964
	(-0.032, -0.024)	(-0.031, -0.023)	
HAALSI-HCAP (South Africa)	-0.047	-0.042	389
	(-0.058, -0.035)	(-0.053, -0.032)	

Notes: All models include survey weights.

Model description: Model 1 regresses domain factor scores on age. Model 2 includes a categorical variable for gender. 95% CI provided in brackets.

Data sources: Data sources: Health and Retirement Study - Harmonized Cognitive Assessment Protocol (HRS-HCAP), HRS 2016 core, National Health and Aging Trends Study (NHATS) - Round 5/2015, the English Longitudinal Study of Ageing-HCAP (ELSA-HCAP), Chile Cognitive Aging Study (Chile-Cog), Mexican Cognitive Aging Study (Mex-Cog), Longitudinal Aging Study in India - Diagnostic Assessment of Dementia (LASI-DAD), and Health and Aging in Africa: A Longitudinal Study in South Africa-HCAP (HAALSI-HCAP).

Supplementary Table 4: Regression coefficients (β) for age from linear models for the orientation domain by HCAP study, N=16,928

Study	Model 1(age)	Model 2 (age and gender)	N
HRS-HCAP (US)	-0.024	-0.024	3347
	(-0.028, -0.020)	(-0.028, -0.020)	
ELSA-HCAP (England)	-0.026	-0.026	1273
	(-0.033, -0.020)	(-0.033, -0.020)	
Mex-Cog (Mexico)	-0.054	-0.054	1256
	(-0.067, -0.041)	(-0.067, -0.042)	
Chile-Cog (Chile)	-0.052	-0.052	1491
	(-0.066, -0.038)	(-0.066, -0.038)	
LASI-DAD (India)	-0.034	-0.032	2964
	(-0.039, -0.028)	(-0.037, -0.027)	
HAALSI-HCAP (South Africa)	-0.052	-0.047	389
	(-0.065, -0.039)	(-0.059, -0.034)	

Notes: All models include survey weights.

Model description: Model 1 regresses domain factor scores on age. Model 2 includes a categorical variable for gender. 95% CI provided in brackets.

Data sources: Data sources: Health and Retirement Study - Harmonized Cognitive Assessment Protocol (HRS-HCAP), HRS 2016 core, National Health and Aging Trends Study (NHATS) - Round 5/2015, the English Longitudinal Study of Ageing-HCAP (ELSA-HCAP), Chile Cognitive Aging Study (Chile-Cog), Mexican Cognitive Aging Study (Mex-Cog), Longitudinal Aging Study in India - Diagnostic Assessment of Dementia (LASI-DAD), and Health and Aging in Africa: A Longitudinal Study in South Africa-HCAP (HAALSI-HCAP).

Supplementary Table 5: Regression coefficients (β) for age from linear models for the executive domain by HCAP study, N=16,928

Study	Model 1(age)	Model 2 (age and gender)	N
HRS-HCAP (US)	-0.064<br>(-0.069, -0.059)	-0.064<br>(-0.069, -0.059)	3347
ELSA-HCAP (England)	-0.069<br>(-0.076, -0.062)	-0.068<br>(-0.076, -0.061)	1273
Mex-Cog (Mexico)	-0.062<br>(-0.072, -0.052)	-0.062<br>(-0.073, -0.052)	1256
Chile-Cog (Chile)	-0.069<br>(-0.081, -0.058)	-0.069<br>(-0.081, -0.057)	1491
LASI-DAD (India)	-0.031<br>(-0.035, -0.026)	-0.030<br>(-0.034, -0.025)	2964
HAALSI-HCAP (South Africa)	-0.042<br>(-0.053, -0.031)	-0.040<br>(-0.050, -0.029)	389

Notes: All models include survey weights.

Model description: Model 1 regresses domain factor scores on age. Model 2 includes a categorical variable for gender. 95% CI provided in brackets.

Data sources: Data sources: Health and Retirement Study - Harmonized Cognitive Assessment Protocol (HRS-HCAP), HRS 2016 core, National Health and Aging Trends Study (NHATS) - Round 5/2015, the English Longitudinal Study of Ageing-HCAP (ELSA-HCAP), Chile Cognitive Aging Study (Chile-Cog), Mexican Cognitive Aging Study (Mex-Cog), Longitudinal Aging Study in India - Diagnostic Assessment of Dementia (LASI-DAD), and Health and Aging in Africa: A Longitudinal Study in South Africa-HCAP (HAALSI-HCAP).

Supplementary Table 6: Regression coefficients (β) for age from linear models of general cognitive function, includes education as a covariate

Study	Model	N
HRS-HCAP (US)	-0.062 (-0.066, -0.057)	3347
HRS (US)	-0.038 (-0.041, -0.036)	9264
NHATS (US)	-0.039 (-0.042, -0.037)	7333
ELSA-HCAP (England)	-0.067 (-0.075, -0.059)	1273
Mex-Cog (Mexico)	-0.057 (-0.066, -0.049)	1256
Chile-Cog (Chile)	-0.053 (-0.063, -0.043)	1491
LASI-DAD (India)	-0.032 (-0.036, -0.028)	2964
HAALSI-HCAP (South Africa)	-0.043 (-0.053, -0.032)	389

Notes: All models include survey weights.

95% CI provided in brackets.

Data sources: Data sources: Health and Retirement Study - Harmonized Cognitive Assessment Protocol (HRS-HCAP), HRS 2016 core, National Health and Aging Trends Study (NHATS) - Round 5/2015, the English Longitudinal Study of Ageing-HCAP (ELSA-HCAP), Chile Cognitive Aging Study (Chile-Cog), Mexican Cognitive Aging Study (Mex-Cog), Longitudinal Aging Study in India - Diagnostic Assessment of Dementia (LASI-DAD), and Health and Aging in Africa: A Longitudinal Study in South Africa-HCAP (HAALSI-HCAP).

Supplementary Table 7: Regression coefficients () for age from linear models of general cognitive function, India, Mexico and South Africa - by literacy status

Study	Model 1 (age)	Model 2 (age and gender)	N
LASI-DAD - Illiterate	-0.029<br>(-0.034, -0.025)	-0.030<br>(-0.034, -0.025)	1724
LASI-DAD - Literate	-0.049<br>(-0.057, -0.042)	-0.050<br>(-0.058, -0.043)	1240
Mex-Cog - Illiterate	-0.041<br>(-0.052, -0.030)	-0.040<br>(-0.051, -0.029)	216
Mex-Cog - Literate	-0.064<br>(-0.072, -0.056)	-0.064<br>(-0.072, -0.056)	1022
HAALSI - Illiterate	-0.033<br>(-0.044, -0.022)	-0.032<br>(-0.043, -0.021)	266
HAALSI - Literate	-0.040<br>(-0.056, -0.024)	-0.040<br>(-0.056, -0.023)	123

Model description: Model 1 regresses general cognitive factor scores on standardized age. Model 2 adjusts for sex. 95% CI provided in brackets.

Data sources: LASI-DAD, Mex-Cog, and HAALSI.

Supplementary Table 8: Heterogeneity in Age-Cognition Associations by Domain, includes education as a covariate

Domain	IV-Weighted CV (%)	Tau
General Cognition	26.4	0.0124
Memory	22.2	0.0082
Language	26.9	0.0074
Orientation	29.7	0.0091
Executive Function	42.7	0.0141

Note:
CV = coefficient of variation (inverse-variance weighted); tau = between-study standard deviation. Both metrics calculated for age coefficients from linear regression models (age, gender, education) across studies.

Supplementary Table 9: Regression coefficients (β) for age from weighted Bayesian linear models of general cognitive function (with measurement error correction)

Study	Model 1 (age)	Model 2 (age and gender)	N
HRS-HCAP	-0.068	-0.069	3347
	(-0.072, -0.064)	(-0.073, -0.065)	
HRS	-0.043	-0.043	9264
	(-0.045, -0.041)	(-0.045, -0.041)	
NHATS	-0.049	-0.049	7333
	(-0.052, -0.046)	(-0.052, -0.046)	
ELSA-HCAP	-0.074	-0.074	1273
	(-0.080, -0.067)	(-0.080, -0.067)	
Mex-Cog	-0.072	-0.073	1256
	(-0.080, -0.065)	(-0.080, -0.065)	
Chile-Cog	-0.068	-0.068	1491
	(-0.075, -0.061)	(-0.075, -0.061)	
LASI-DAD	-0.040	-0.039	2964
	(-0.045, -0.035)	(-0.044, -0.034)	
HAALSI-HCAP	-0.048	-0.045	389
	(-0.059, -0.038)	(-0.055, -0.035)	
